# Parental Influence on Child Mental Health Post-Hurricane Harvey

**DOI:** 10.1007/s40653-023-00554-w

**Published:** 2023-05-16

**Authors:** Mary B. Short, Savannah Kaye, Cory Knight, Alexa Riobueno-Naylor, Betty Lai, Sara Elkins, Thomas Schanding, Steven L. Bistricky

**Affiliations:** 1 Department of Clinical, Health, and Applied Sciences, University of Houston-Clear Lake, Houston, TX United States; 2 Department of Counseling, Developmental, and Educational Psychology, Boston College, Chestnut Hill, United States; 3 Department of Educational and Counseling Psychology, University of British Columbia, British Columbia, Canada; 4 Department of Psychology, University of Colorado, Colorado, United States

**Keywords:** Hurricane, Natural Disaster, Parenting, Psychosocial Functioning, Internalizing Symptoms, Externalizing Symptoms

## Abstract

**Purpose:**

Many children who face natural disasters experience significant mental health consequences. Parents play a prominent role in the likelihood of child mental health outcomes after a weather-related disaster. This study aimed to examine the relationship between parent risk factors and children’s psychological well-being post Hurricane Harvey.

**Methods:**

Parents (n = 140) completed a survey that measured hurricane exposure, parental depression and anxiety, parenting behaviors, and assistance given and received during or after Hurricane Harvey. Additionally, parents were asked to complete questionnaires assessing one of their children’s post-disaster psychosocial functioning and distress.

**Results:**

Results indicated that heightened parent anxiety was significantly associated with an increased risk for emotional symptoms, conduct problems, and hyperactivity-inattention symptoms in children. Additionally, inconsistency in parental discipline was significantly associated with an increased risk of child conduct problems. Further, higher numbers of assistance types received by parents—a proxy indicator of resource loss—was associated with higher child emotional distress scores.

**Conclusions:**

Broader systems-level interventions that address parents’ physical and emotional needs may help mitigate maladaptive reactions in children and facilitate greater post-disaster psychosocial adjustment.

In August and September 2017, Hurricane Harvey unleashed 33 trillion gallons of rain on Texas and Louisiana (Shultz & Galea, 2017), resulting in widespread flooding for many weeks. Thirteen million people were directly impacted by the storm, which caused massive financial and property losses, prolonged life disruption, fear, and trauma (Amos Nwankwo et al., 2021).

## Resources, Disaster Impacts, Risk, and Protection

Conservation of Resources (COR) theory (Hobfoll, 1989) can help frame the impacts of disasters and how communities, families, and individuals respond. COR theory emphasizes that humans are motivated to acquire, protect, and replace resources that promote survival, effective functioning, and well-being. Resources include objects (e.g., house, car), conditions (e.g., employment, stable mental health), personal characteristics (e.g., coping skills) or energies (e.g., time) “that are valued in their own right, or that are valued because they act as conduits to the achievement or protection of valued resources” (Hobfoll, 2001, p. 339). Resources can be gained or depleted (e.g., social connections and self-regulatory energy) and are often interdependent. Therefore, depletion or loss in one domain (e.g., job layoff) can spiral into losses across other domains (money, house, food, childcare, transportation, routine/stability, perceived self-efficacy). In this study, COR theory helps frame an understanding of how Hurricane Harvey affected children directly and indirectly through their parents.

Across the age spectrum, weather event-related disasters can greatly impact health, with long-term effects often worse than effects that occur during the disaster’s most acute stage (Shultz & Galea, 2017). Both adults and children are at increased risk for experiencing functionally impairing levels of distress and mood disorders after a disaster (see La Greca, 2013 and Lai et al., 2013 regarding Hurricane Andrew and Hurricane Ike, respectively). People commonly experience symptoms of depression, anxiety, and posttraumatic stress as well as other internalizing and externalizing problems (see Goldmann & Galea 2014 for review). However, impacts of hurricanes and other disasters are not equally distributed across the population. Thus, better understanding potentially mutable factors (i.e., resources) that confer differential risk from the deleterious effects of disasters could inform governmental relief agencies, mental health systems, and the public on how best to mitigate these effects. Although it is important to recognize extant research on effects related to past trauma exposure (Sayed et al., 2015), preparedness from past disaster experience (see Bistricky et al., 2019; Shrira et al., 2014 regarding Hurricanes Harvey and Sandy, respectively), socioeconomic stressors and access to resources (e.g., Hobfoll et al., 2006), and trait resilience (see Long et al., 2020 regarding Hurricane Harvey), the present study focused on a different collection of factors conceptually related to children’s risk or protection from mental health problems. Many of these conceptually plausible relations have not received adequate empirical examination on their own, much less in combination with each other. Thus, the present research sought to help address this.

### Exposure

Level of hurricane/flood exposure is among the most heavily researched risk factors and has been found to be a particularly strong predictor of adult and child psychological well-being (Bistricky et al., 2019; see La Greca et al., 1998 regarding Hurricane Andrew; Long et al., 2020; see Vernberg et al., 1996 regarding Hurricane Andrew). Given how reliable and robust effects of exposure are in the literature, studies should account for degree of exposure when examining other potential risk and protective factors. Hurricane/flood exposure severity is typically assessed in terms of perceived life threat, actual life threat, and immediate loss/disruption (e.g., losing a family member, loss of housing). Overall, level of exposure has been found to predict the severity of depressive symptoms as well as anxious distress in adults and children (see Bokszczanin 2008 regarding 1997 flooding in southern Poland; La Greca et al., 1998; see Lai et al., 2013 regarding Hurricane Katrina; Long et al., 2020).

In children, disaster exposure’s influence on mental health may be partially mediated through social support from parents, peers, and teachers, which can be significantly disrupted after a disaster (see Hardin et al., 1994 regarding Hurricane Hugo; Lai et al., 2015, 2018a, b> regarding Hurricane Katrina). Thus, in the present study we expected greater exposure would be related to greater depression and anxiety in parents, more disruption in parenting behaviors, and greater emotional symptoms in children.

### Parent Symptoms and Parenting Behaviors

Parents’ post-disaster mental health and parenting behavior can also influence children’s psychological well-being. For example, a study by Bokszczanin et al. (2008) found that children who experienced excessive parental control and higher levels of exposure following a massive flood had increased symptoms of distress. Notably, children and parents have reciprocal roles that can contribute to the development of post-disaster psychopathology (Self-Brown et al., 2017). As parents attempt to cope, meeting the needs of a child is an additional stressor that can be exacerbated by other variables, such as a child’s developmentally related verbal and self-regulation abilities or a single parent’s potential lack of caregiving support. Resulting strain can increase their risk for psychopathology (La Greca et al., 1998) and negatively affect parenting practices (Berg-Nielsen et al., 2002). At the same time, children’s behavior and psychological well-being can be affected by family dynamics, parental support, and parental mental health after a disaster (Lai et al., 2018a, b; Spell et al., 2008; (Wasserstein & La Greca, 1996; Wasserstein & Greca, 1998 regarding Hurricanes Andrew and Katrina). Per COR theory, these factors can be thought of as condition resources that may be maintained, depleted, or lost.

#### Anxiety and Depression

Parental psychopathology has been found to predict child internalizing and externalizing mental health problems (Spell et al., 2008). For example, children’s risk for developing symptoms of anxiety and depression increases significantly when parents themselves begin to exhibit these symptoms. This is likely influenced by emotional, cognitive, and behavioral contagion (Abela et al., 2009; Joiner & Katz, 1999; Paz et al., 2022), as children often mimic their parents’ distress after a disaster, and children can also be stressed by their parents’ distress (Hoven et al., 2009; La Greca et al., 1998). Therefore, parents who are not coping effectively with the stressors post-disaster may also influence children’s psychological well-being (Hoven et al., 2009). In turn, children’s symptoms of depression and anxiety can partly maintain parents’ symptoms, forming a maladaptive cycle (Jalmsell et al., 2010). In line with extant theory and research, we expected that parent anxiety and depression symptoms would be associated with parallel symptoms in children. We also anticipated that greater levels of symptoms in parents could result in child distress manifesting in externalizing behaviors, with greater conduct problems and hyperactivity-inattention symptoms (Deault, 2010; Kashdan et al., 2004; Spell et al., 2008). Given parental anxiety can transmit cognitive uncertainty, anxious arousal, and a sense of helplessness to children, we thought it might have an accentuated, pervasive effect across children’s behavioral health.

#### Positive Parenting Behaviors and Parental Discipline

Relatedly, during stressful times, parents’ balanced involvement, support, and maintaining reliable routines and contingency management all bolster children’s ability to cope and regulate their emotions and behavior (Bistricky et al., 2021; Bokszczanin 2008; Wasserstein & La Greca, 1998). Two particularly important contingency-focused parenting behaviors are how parents respond to a child’s desired prosocial behaviors and how parents respond to a child’s misbehaviors. *Positive parenting* alludes to how regularly a parent acknowledges and praises a child’s prosocial behavior, positive reinforcement that typically begets more prosocial behavior (Rudy & Grusec, 2020; Spinrad & Gal, 2018). Conversely, *inconsistent discipline* refers to how frequently parents do not follow through on consequences for misbehavior. When the misbehavior produces desirable outcomes for the child (e.g., otherwise inaccessible power or materials) that outweighs occasional disciplinary consequences, the variable ratio reinforcement can result in continued misbehaviors and conduct problems (Murray et al., 2010).

For praise and discipline to be administered effectively, parents must mobilize self-regulation resources to sustain their own selective attention, motivation, and sustained effort (Sanders et al., 2019). With a region-wide flood event such as Hurricane Harvey, even the least affected individuals experienced disruptions and shifts in important daily routines. Under these conditions, children may test boundaries and act out related to disaster-induced environmental uncertainties (e.g., school and daycare closing indefinitely) and attempt to ascertain if the parental contingencies they are accustomed to are still reliably in place (see Norris et al., 2002; Pfefferbaum, Pfefferbaum & Norris, 2010 regarding Hurricane Katrina). Also, a child who is distressed in the aftermath of disaster may seek out contingent parental praise through prosocial behaviors, such as completing chores and seeking out appropriate ways they might help the parent or family. For a parent, providing praise may be a relatively low investment of their own self-regulation resources and it could result in motivational resource-replenishing positive emotional experiences, such as a sense of connectedness and pride in one’s child.

By comparison, providing consistent discipline may require greater self-regulation and result in less unambiguous reinforcement of this effective parenting behavior. For example, immediately following discipline a child could become upset and/or escalate acting out. This might require the parent to invest even more attention, effort, and emotional resources to console their child and administer consequences consistently, but also to regulate the parent’s own additional distress from these interactions. When these challenges are added to other post-disaster stressors, it can simply be easier for parents to accommodate child misbehaviors (Elkins et al., 2021), inadvertently reinforcing them. Thus, the present study sought to examine whether positive parenting and inconsistent discipline after a disaster would relate to greater emotional distress, emotional symptoms, conduct problems, and hyperactivity-inattention in children. We anticipated that positive parenting might support children’s sense of hope, stability, and agency, thus, reducing rates of anxious and depressive symptoms in these children. Conversely, we expected that inconsistent discipline would be linked to conduct problems.

### Assistance

#### Received

Receiving assistance from family, friends, and relief agencies after a disaster can be considered a needed and useful infusion of resources (Layne et al., 2021). At the same time, outside resource infusions may not fully compensate for losses of key resources. Further, receiving higher amounts of assistance may correspond with greater resource loss and resulting distress (Bistricky et al., 2019). This loss and distress might, in part, impact children’s mental health by interacting with previously described factors. For example, parents’ self-regulation resources can be significantly taxed in the aftermath of disaster (Sanders et al., 2019), resulting in greater parental anxiety, depression, and less-effective parenting and support for their children (Bokszczanin, 2008; La Greca et al., 1998). Resource losses, represented by greater need for assistance, can also impact children’s behavioral health through destabilization within family dynamics. For example, perceived family conflict can increase anxiety and externalizing behaviors in children (Wasserstein & La Greca, 1996, 1998). Thus, conceptualizing that the amount of assistance parents received from family, friends, and relief agencies would reflect amount of resource depletion, we expected that greater assistance would correlate with greater symptoms of psychopathology and misbehavior in children in our study.

#### Provided

Disaster survivors often provide assistance (i.e., resources) to others in their communities (Bistricky et al., 2019), but findings are mixed on whether amount of post-disaster assistance provided correlates with symptoms of psychopathology. In fact, it may depend on whether providing assistance causes stress or serves as a reminder of the experience. Further, it may depend on whether this relation is assessed cross-sectionally or longitudinally (see Balderas-Badawi et al., 2023 regarding Hurricane Harvey). In our study, we had no specific hypothesis, but we sought to account for assistance provided while examining other potential risk and protective factors.

### Minoritized Status

In the aftermath of disasters, women are particularly vulnerable to distress and depression, in part due to rises in intimate partner violence experienced (see Bell et al., 2016 for review). Research also shows that racial and ethnic communities often experience disproportionally elevated impacts from disasters while also experiencing disparities in access to assistance and quality mental health care (Benevolenza & DeRigne, 2019; Bolin & Kurtz, 2018; Norris & Alegria, 2005). Although the substantial collective support within these minoritized (i.e., subordinated, oppressed) groups might partly buffer stress associated with minoritized status (cf. Meyer, 2003; Shepherd et al., 2018), we anticipated that children who identify as girls and/or whose reporting parent/caregiver identifies with a minoritized racial-ethnic group might show worse mental health outcomes in the wake of the hurricane.

## Summary of the Present Study

There is limited understanding about the combined effects of disaster exposure, parental mental health, parenting behaviors, post-disaster assistance, and minoritized status on children’s mental health after a weather-related disaster. This study aimed to better understand how these factors relate to children’s emotional distress, depressive and anxious emotional symptoms, hyperactivity-inattentiveness, and conduct problems. As previously reviewed, we hypothesized that lower disaster exposure, better parent mental health, and effective praise and discipline with children during the aftermath of Hurricane Harvey would correlate with more positive outcomes.

## Methods

### Participants

Participants were recruited via online advertisements, an email to all University students and employees, the student research pool, and snowball electronic communications (Bistricky et al., 2019). Participants completed one assessment at 1–3 months following Hurricane Harvey. The initial sample included 1,084 adult respondents. Respondents who answered affirmatively to the question, “Do you have children in the home (2–17 years old) who we could ask you a few questions about?” and provided information about their child’s age and gender were included in the sample. The final cross-sectional sample of parents and children included 140 adults (77.6% women) with at least one child between the ages of 2–17 years (52.9% girls).

Descriptive statistics for all study variables are reported in Table 1. Parents were between 18 and 64 years old, with a majority (66.4%, *n* = 93) of parents falling between the ages of 25–44. Children were between the ages of 2–17, including 35 children (25.0%) who were 2–4 years old, 45 children (32.1%) who were 5–10 years old, and 60 (42.9%) 11-17-year-old children. Parents were racially and ethnically diverse, with 54.3% (*n* = 76) identifying as white, 27.1% (*n* = 38) identifying as Hispanic/Latinx, 10.7% (*n* = 15) identifying as Black/African American, 5.0% (*n* = 7) identifying as Asian/Pacific Islander, and 2.9% (*n* = 4) identifying as “other.” A majority of families (71.4%, *n* = 100) had an income below $75,000. Most (64.3%, *n* = 97) parents completed at least some college and nearly all (98.6%, *n* = 138) were either currently employed or a student.

**Table 1:** Variable Descriptives and Frequencies (*N* = 140)

Hurricane Exposure	*M (SD)*
Actual Life Threat (0–6)	0.47 (0.86)
Immediate Loss/Disruption (0–10)	1.84 (1.90)
	*N (%)*
Perceived Life Threat	29 (20.7)
Parent Mental Health	*M (SD)*
Depression (0–24)	7.22 (6.24)
Anxiety (0–21)	7.44 (6.09)
	*M (SD)*
Positive Parenting (1–15)	13.02 (2.81)
Inconsistent Discipline (1–15)	6.66 (2.75)
Post-Hurricane Assistance ^1^	* N (%)*
Received Assistance from Agency Only	14 (10.0)
Received Assistance from Family/Friends Only	25 (17.9)
Received Assistance from Agency and Family/Friends	20 (14.3)
Provided Assistance to Others	112 (80.0)
	*M (SD)*
Strengths and Difficulties Emotional Symptoms (0–10)	3.36 (2.90)
Strengths and Difficulties Conduct Problems (0–10)	2.09 (2.09)
Strengths and Difficulties Hyperactivity-Inattention (0–10)	4.60 (2.66)
Emotional Distress (21–84)	35.63 (14.77)

^1^ Parents were asked if they received or provided assistance in the aftermath of the hurricane, what types of assistance they received and/or provided, and where they received assistance from. The range of types of assistance received was 0–10. Fifty-nine families received assistance. The average number of types of assistance from any source received by the families was 4.54 (*SD* = 3.37). The range of types of assistance provided was 0–8. Of the 112 families who provided assistance, the average number of different types of assistance provided was 5.23 (*SD* = 1.75)

### Procedures

The study was approved by the Institutional Review Board for the University where the study was conducted. Convenience sampling was utilized. Participants were recruited in four ways, including website advertisements (i.e., Facebook, parenting forums), snowball emailing methods (asking contacts to email the research opportunity to their contacts), using a university participant pool, and emailing contacts. The participant pool was associated with a university in the gulf-coast of Texas with a large proportion of non-traditional students who were also parents. Participants, who signed up through the participant pool, received extra credit for their time. All other participants were offered to be entered into a raffle for a $50 gift card. Inclusionary criteria included being English-speaking adults (over 18) who experienced Harvey. All responses possessed a unique identifier in order to prevent duplication of the survey.

During recruitment, potential participants were provided with a link to informed consent followed by the survey, which was housed on the secure Qualtrics platform. The adult participants completed all survey questions. A majority of the survey questions were related to their own experiences during and after Hurricane Harvey. Further, the participants were asked if they had a child. If they did not have a child, the study ended. If they did have a child, they were asked to complete several questionnaires regarding one of their children. For parents with more than one child, participants were asked to focus on one child and choose the child who was most affected by Hurricane Harvey, as the most affected child is the child in most need of parental support. Further, focusing on one child was expected to decrease confusion for parents when completing the surveys. Surveys were completed within approximately 60 min. Data were collected from September 29-November 30, 2017 (4–10 weeks after August 25th landfall of Hurricane Harvey).

### Measures

All measures in this study were parent-reported, including assessments of child outcomes. Parents provided reports on demographics (self and child), parent Hurricane Harvey exposure, depression, anxiety, parenting characteristics, child psychosocial strengths and difficulties, child emotional distress, and whether they provided or received assistance in the aftermath of the hurricane.

#### Parent-Focused Measures

##### **Parent and Child Demographics**

Dummy coded variables were created for child gender identity and parent race/ethnicity. Child gender included girl (coded as 1) and boy (coded as 0). Data related to non-binary and transgender identities were not collected. Race/Ethnicity was recoded dichotomously to compare minoritized (Hispanic/Latinx, Black/African American, Asian/Pacific Islander, other; *n* = 64; coded as 1) versus white (*n* = 76; coded as 0) participants.

##### **Parent Hurricane Exposure**

Parent exposure to Hurricane Harvey was measured using the 21-item Hurricane-Related Traumatic Experiences Questionnaire–Revised (HURTE-R; La Greca et al., 1996; Vernberg et al., 1996). The measure includes three subscales which evaluate different components of exposure. The actual life threat scale is a sum of 6 items (yes/no) regarding exposure types of events that may have caused threat of life or serious harm to the individual (e.g., “Did you get hit by anything falling or flying during the Hurricane?”). The perceived life threat scale includes one yes/no item which asked, “At any time during the Hurricane, did you think you might die?” The immediate loss/disruption scale was calculated by summing 10 yes/no questions about loss/disruption in 2 months following the hurricane (e.g., “Did you move to a new place because of the hurricane?”).

##### **Parent Depression**

The Patient Health Questionnaire-8 (PHQ-8; Kroenke et al., 2009) was used to assess levels of parent depression. The PHQ-8 consists of eight items that are used to assess depression symptoms experienced in the past two weeks, and one item that assesses the impact of the endorsed symptoms on the person’s life. The eight items that are summed to create the total depression severity score utilize a 4-point response scale (0 = “not at all” to 3 = “nearly every day”). The PHQ-8 total score ranges from 0 to 24, with higher scores indicating worse depression symptoms and a score of 10 or above indicating possible depression (Shin et al., 2019; Kroenke et al., 2009). The PHQ-8 has demonstrated excellent internal consistency in previous studies (*α* = 0.88; Shin et al., 2019). Excellent internal consistency was also demonstrated in this sample (*α* = 0.91).

##### **Parent Anxiety**

The Generalized Anxiety Disorder Screener-7 (GAD-7; Spitzer et al., 2006) was used to assess parent anxiety symptoms. The GAD-7 consists of seven items which assess anxiety symptoms in the past two weeks. Participants responded to each item using a 4-point response scale (0 = “not at all” to 3 = “nearly every day”). Responses were summed to compute a total anxiety severity score. The GAD-7 total score ranges from 0 to 21, with higher scores indicating worse anxiety symptoms and a score of 10 or higher indicating possible anxiety. The GAD-7 has demonstrated excellent internal consistency (*α* = 0.92; Spitzer et al., 2006) in previous studies as well as in the current sample (*α* = 0.94).

##### **Parenting Behaviors**

Parenting was assessed via two of the three subscales of the Alabama Parenting Questionnaire-9 (APQ-9; Elgar et al., 2007). The full scale includes nine questions that are answered using a 1–5 Likert scale (1= “never” to 5 = “always”). The positive parenting and inconsistent discipline subscales were utilized in the current study. The poor supervision subscale was excluded given that questions were not developmentally appropriate for younger children. The positive parenting subscale is the sum of three items, including “you let your child know when he/she is doing a good job with something.” The inconsistent discipline subscale assessed parent agreement with three questions including, “you threaten to punish your child and then do not actually punish him/her.” Acceptable internal consistency has been demonstrated across subscales (Liang et al., 2021). In the current sample, internal consistencies for the positive parenting and inconsistent discipline subscales were *α* = 0.94 and *α* = 0.77, respectively.

##### **Assistance Received**

Parents were asked whether they received assistance “during Hurricane Harvey and/or its aftermath,” where they received the assistance from (from an agency [e.g., FEMA, Red Cross, a church] or family/friends), and what type of assistance they received from either source (evacuation services, shelter, food/water, clothing, demolition assistance, transportation, household items, childcare, financial, and other). A summary variable was created to capture the number of different types of assistance that parents received from the agency or family friends. The summary variable represented the number of types of support a parent received; thus, if a parent received 5 types of help, they would receive a 5. Scores had a possible range of 0–10 types.

##### **Assistance Given**

Parents were also asked about whether they provided any of the following 8 types of assistance to others: daily needs (e.g., food, water, clothing, or other material goods), emotional support, specific skills related to their education or training, temporary shelter for people displaced by the storm and flooding, goods distribution, instrumental help (giving rides, watching others’ children, doing others’ laundry, etc.), tearing down and throwing out flooded parts of homes/buildings and/or rebuilding damaged homes/buildings, or other. A summary variable included the total sum of types of assistance parents provided. Again, the summary variable represented the number of types a parent provided; thus, if a parent provided 5 types of help, they would receive a 5. Scores had a possible range of 0–8.

#### Child-Focused Measures

##### **Child’s Psychosocial Strengths and Difficulties**

Parents assessed their child’s psychosocial functioning via the Strengths and Difficulties Questionnaire (SDQ; Goodman, 1997). The SDQ consists of 25 items answered on a 3-point Likert scale (0 = “not true”, 1= “somewhat true,” 2= “certainly true”) that are divided into five subscales: (1) emotional symptoms; (2) conduct problems; (3) hyperactivity-inattention; (4) peer problems; and (5) prosocial behavior. The first three scales were utilized in the present analyses. Subscales include five questions and are scored by summing each question (possible range per subscale between 0 and 10). Higher scores indicate heightened difficulties related to emotions, conduct, or hyperactivity-inattention.

Three different versions of the SDQ have been validated in the following age groups: 2–4 years, 5–10 years, and 11–17 years. Versions are slightly modified to be more developmentally appropriate to the age group (e.g., “rather solitary, prefers to play alone” for ages 2–4 and for ages 11–17 “would rather be alone than with other youth”). The SDQ has demonstrated satisfactory internal consistency across age groups (Goodman, 2001). In the current sample, the internal consistency of the total score was strong across age groups: *α* = 0.82 (2–4 years), *α* = 0.80 (5–10 years), and *α* = 0.84 (11–17 years).

##### **Child Emotional Distress**

Parents were asked about their child’s emotional distress using the Pediatric Emotional Distress Scale (PEDS; Saylor et al., 1999). The PEDS includes 21-items answered on a 4-point Likert scale (1= “almost never” to 4= “very often”). Parents were asked about a child with whom they experienced a traumatic event. The measure includes three subscales which are summed into a total score (range = 21–84): anxious, fearful, and acting out. Higher scores indicate more severe emotional distress. A majority (*n* = 17) of the items are related to general child behaviors (e.g., “gets frustrated too easily”) while the remaining four items ask about symptoms related to trauma exposure (e.g., “seems fearful of things that are reminders of the event[s]”). The PEDS total score has demonstrated acceptable internal consistency (Saylor et al., 1999). In the current sample, the total score demonstrated excellent internal consistency (*α* = 0.96).

### Analytic Plan

Analyses were conducted with SPSS Statistics (Version 27). Descriptive statistics were evaluated regarding demographics (parent and child age, parent race/ethnicity, family income, parent education, and parent employment status), parent Hurricane Harvey exposure, parent mental health, parenting behaviors, whether parents received and/or provided post-hurricane assistance, child psychosocial difficulties, and child emotional distress. To examine relationships between variables, we used bivariate correlation analysis.

Associations between parental factors and child mental health were assessed using linear regression analysis. With child psychosocial difficulties and child emotional distress as the continuous outcomes, we evaluated the possible influence of the following factors: parent experiences of actual life threat during the hurricane, parent experiences of perceived life threat during the hurricane, parent experiences of immediate loss/disruption post-hurricane, the number of agency assistance types parents received (possible range: 0–10), the number of non-agency assistance types parents received (possible range: 0–10), the number of assistance types parents provided to others (possible range: 0–8), parent depression post-hurricane, parent anxiety post-hurricane, positive parenting behaviors, inconsistent parental discipline, female gender (child), and whether the parent was a member of a minoritized racial/ethnic group.

## Results

### Parent Hurricane Exposure

Parents endorsed an average of 0.47 (*SD* = 0.86) incidents of actual life threat out of a possible 6, including thinking someone might die during the hurricane (61.4%, *n* = 86) and thinking they may be hurt badly during the hurricane (30.0%, *n* = 42). About one-fifth (20.7%, *n* = 29) of parents endorsed an experience of perceived life threat. The average number of types of immediate loss/disruption endorsed by parents was 1.84 (*SD* = 1.90) out of a possible 10, including experiencing flooding during the hurricane (65.7%, *n* = 92) and having trouble getting enough food and water after the hurricane (42.1%, *n* = 59). Generally, greater exposure corresponded with higher levels of child difficulties, parent symptoms, and inconsistent discipline by parents (see Table 2). Notably, exposure was *not* associated with positive parenting behaviors. Also, self-identifying with a minoritized racial-ethnic group was weakly but significantly associated with greater perceived life threat.

**Table 2:** Correlations Among Study Variables (*N* = 140)

Variables	1	2	3	4	5	6	7	8	9	10	11	12	13	14	15
**1**) Emotional Symptoms (CSD)	--														
**2**) Conduct Problems (CSD)	0.57***	--													
**3**) Hyperactivity- Inattention (CSD)	0.54***	0.48***	--												
**4**) Child Emotional Distress	0.60***	0.54***	0.49***	--											
**5**) Actual Life Threat	0.32***	0.29***	0.19*	0.43***	--										
**6**) Perceived Life Threat	0.14	0.08	0.09	0.20*	0.30***	--									
**7**) Immediate Loss /Disruption	0.24**	0.18*	0.17	0.36***	0.62***	0.38***	--								
**8**) Number of Agency Assistance Types Received	0.28***	0.22***	0.23**	0.34***	0.47***	0.27**	0.58***	--							
**9**) Number of Types of Assistance Provided	0.21*	0.24*	0.12	0.30**	0.32***	0.18	0.34***	0.10	--						
**10**) Parent Depression	0.27**	0.16	0.18*	0.37***	0.36***	0.21*	0.50***	0.26**	0.14	--					
**11**) Parent Anxiety	0.40***	0.29***	0.29***	0.38***	0.33***	0.28**	0.43***	0.23**	0.24*	0.80***	--				
**12**) Positive Parenting	− 0.07	− 0.11	0.02	− 0.15	0.05	0.06	0.10	0.07	0.05	− 0.08	− 0.12	--			
**13**) Inconsistent Discipline	0.21*	0.38***	0.06	0.26**	0.35***	0.15	0.23**	0.22*	0.28**	0.07	0.07	0.16	--		
14) Child Gender ¹	0.06	0.07	− 0.04	0.09	0.12	− 0.01	0.00	− 0.03	0.05	0.05	0.05	0.07	0.11	--	
**15**) Race/Ethnicity ¹	0.20	0.05	− 0.05	− 0.08	0.13	0.17*	0.09	0.01	0.05	0.15	0.04	− 0.04	0.16	− 0.02	--

*p < .05. ** *p* < .01. ***p < .001. ¹ Categorical variables were assessed using Pearson correlation coefficient. Gender and race/ethnicity were categorically recoded, where 1 = female and 0 = male and 1 = minoritized race/ethnicity and 0 = white. *Note*: CSD = Child Strengths and Difficulties Questionnaire.

### Parent Assistance

Fifty-nine parents received assistance, including those who received agency assistance only (10%, *n* = 14), assistance from family/friends only (17.9%, *n* = 25), and those who received assistance from both sources (14.3%, *n* = 20). The average number of types of assistance from any source received by the 59 families was 4.54 (*SD* = 3.37). Of the 59 families who received assistance, the most commonly received assistance type was food and water, (69.5%, *n* = 41), followed by shelter (54.2%, *n* = 32). Of the 34 parents who received assistance from an agency, the most commonly received agency assistance types included financial support (79.4%, *n* = 27), food and water (67.6%, *n* = 23), household items (64.7%, *n* = 22) and shelter (61.8%, *n* = 21). Of the 45 parents who received assistance from family/friends, the most frequently received assistance included food and water (77.8%, *n* = 35), financial support (62.2%, *n* = 28), shelter (62.2%, *n* = 28), transportation (46.7%, *n* = 21), and evacuation services (46.7%, *n* = 21). Amount of assistance received positively correlated with level of hurricane/flood exposure, and particularly strongly with immediate loss/disruption. A majority (80%, *n* = 112) of families provided assistance to others, including providing emotional support (76.4%, *n* = 107) and materials to meet daily needs (e.g., food, water; 75%, *n* = 107). Assistance received and provided did not vary by child gender or by parent minoritized group identity status.

### Parenting and Parent Mental Health

Parents scored an average of 13.02 (*SD* = 2.81) on the positive parenting subscale and an average of 6.66 (*SD* = 2.75) on the inconsistent discipline scale (range of 5–15 for both scales). A majority of parents (59.2%, *n* = 77) indicated they “always” let their child know when they are doing a good job with something, while half (50.0%, *n* = 65) indicated they “never” allow their child to talk them out of being punished after their child has done something wrong. Parents’ self-reported symptoms of depression and anxiety indicated that 32.1% (*n* = 45) had symptom levels consistent with a probable depressive disorder, while 35.7% (*n* = 50) reported symptom levels consistent with a probable anxiety disorder.

### Child Psychosocial Difficulties

Linear regression models were used to examine potential risk factors for emotional symptoms, conduct problems, and hyperactivity-inattention symptoms in children (see Table 3). Predictors included parent hurricane exposure, frequency of post-hurricane assistance received and provided, parent depression and anxiety, parenting behaviors, and child demographics (gender and race/ethnicity). The model for children’s emotional symptoms was significant (*F* (11, 91) = 3.39, *p* < .001) with an adjusted *R*^2^ = 0.21. Similarly, the models for conduct problems and hyperactivity-inattention symptoms in children were also significant (conduct problems: *F* (11, 91) = 3.55, *p* < .001, adjusted *R*^2^ = 0.22; hyperactivity-inattention symptoms: *F* (11, 91) = 2.20, *p* = .021, adjusted *R*^2^ = 0.11).

**Table 3:** Regression Models: Predicting Child Outcomes

Variables	Emotional Symptoms		Conduct Problems		Hyperactivity- Inattention		Emotional Distress		Resilience	
	*β*	*SE*	*β*	*SE*	*β*	*SE*	*β*	*SE*	*β*	*SE*
Actual Life Threat	0.42	0.42	0.13	0.30	0.06	0.41	2.70	1.77	-0.07	0.10
Perceived Life Threat	-0.23	0.72	-0.55	0.52	-0.55	0.70	0.44	3.04	-0.20	0.18
Immediate Loss/Disruption	0.02	0.24	-0.05	0.18	0.01	0.23	-0.11	1.02	-0.01	0.06
Number of Assistance Types Received	0.14	0.11	-0.12	0.08	0.18	0.11	1.11	0.47*	0.01	0.03
Number of Types of Assistance Provided	0.09	0.17	0.07	0.12	0.07	0.17	0.92	0.72	-0.02	0.04
Parent Depression	-0.09	0.07	-0.07	0.05	-0.07	0.07	0.58	0.31	0.02	0.02
Parent Anxiety	0.24	0.07**	0.14	0.05*	0.21	0.07*	0.32	0.30	0.01	0.02
Positive Parenting	-0.05	0.10	-0.10	0.08	0.13	0.10	-0.17	0.43	0.01	0.02
Inconsistent Discipline	0.12	0.11	0.28	0.08***	-0.08	0.10	0.68	0.45	0.02	0.03
Child Gender (Female)	0.11	0.55	0.05	0.40	-0.43	0.54	1.94	2.34	0.17	0.12
Race/Ethnicity (Minority)	0.02	0.58	-0.15	0.42	-0.08	0.56	-4.87	2.45	-0.11	0.13
Variance Explained	*R*^2^ =. 29		*R*^2^ =. 30		*R*^2^ =. 21		*R*^2^ =0.46		*R*^2^ =. 13	

*Note.* Emotional Symptoms Model: *F* (91, 102) = 3.39, *p* < .001; Conduct Problems Model:  *F* (91, 102) = 3.55, *p* < .001; Hyperactivity- Inattention Model: *F* (91, 102) = 2.20, *p* = .021; Emotional Distress Model:  *F* (91, 102) = 7.03, *p* < .001; Resilience Model: *F* (64, 75) = 0.83, *p* =. 61

*p < .05. ** *p* < .01. ***p < .001.

Examining individual risk factors, parent anxiety was significantly associated with an increased risk for emotional symptoms, conduct problems, and hyperactivity-inattentive symptoms in children (emotional symptoms: *β* = 0.24, *SE* = 0.07, *t* = 3.35, *p* = .001; conduct problems: *β* = 0.14, *SE* = 0.05, *t* = 2.64, *p* = .010; hyperactivity-inattention symptoms: *β* = 0.21, *SE* = 0.10, *t* = 3.13, *p* = .002). In addition, higher scores on the inconsistent discipline subscale were associated with a heightened risk of child conduct problems (*β* = 0.28, *SE* = 0.08, *t* = 3.58, *p* < .001). Lastly, receiving more assistance from agencies and/or families and friends was associated with increased emotional distress in children (*β* = 1.11, *SE* = 0.47, *t* = 2.40, *p* = .018).

### Child Emotional Distress

The multiple linear regression model examining factors that increased the risk for child emotional distress scores, capturing child anxiety, fearfulness, and acting out, was also significant (*F* (11, 91) = 7.03, *p* < .001) with an adjusted *R*^2^ = 0.46. The greater the number of assistance types parents received (*β* = 1.11, *SE* = 0.47, *t* = 2.40, *p* = .018) the higher child emotional distress scores were.

## Discussion

With the frequency and intensity of weather-related disasters increasing, understanding disasters’ impact on parents’ and children’s mental health is crucial. The current study builds upon previous disaster exposure literature by examining how parental variables relate to the functioning of children in the aftermath (1–3 months post-disaster) of a major hurricane and flood. Similar to results from Hoven and colleagues (2009) and La Greca et al. (1998), parents’ experiences of anxiety and depression were correlated with their children’s emotional symptoms, hyperactivity/inattention, and emotional distress at 1–3 months post-hurricane. Parental anxiety was also correlated with a youth’s conduct problems. While previous literature has highlighted the relationship between parental depression and childhood conduct problems (Gross et al., 2008; Hails et al., 2018; Marchand et al., 2002), parental depression was not predictive of child conduct problems in the current sample.

Additionally, results of this study provide further support for the Conservation of Resources theory. In examining the correlations among parental losses related to Hurricane Harvey, parents’ reports of immediate loss/disruption, as well as receiving various types of assistance from agencies, were correlated with youth’s mental health and conduct. Further, parents’ experiences of disruption and loss during this event were predictive of youth’s emotional distress, as measured by the PEDS, specifically for the number of assistance types received. Other types of losses, such as actual/perceived threats to life were not predictive of youth’s mental health functioning or conduct at 1–3 months post-hurricane. This may be due to the small number of individuals reporting actual or perceived threats to life. In addition, greater receipt of assistance was related to higher use of inconsistent discipline following the hurricane, and higher inconsistent discipline predicted greater youth emotional and behavioral concerns. Consistent with COR theory, as physical resources are depleted in the aftermath of a disaster, parents may engage in more reactive parenting practices (compared to proactive) that contribute to reinforcement of emotional and conduct related symptoms in children (Elkins et al., 2021).

Study findings regarding parenting practices are particularly noteworthy. Within this sample, positive parenting practices were neither correlated with nor predictive of child mental health and conduct. While parenting practices may act to support children during a weather-related disaster (Bokszczanin, 2008; Wasserstain & La Greca, 1998; Wasserstein & La Greca 1996), positive parenting alone may not be enough to protect youth from experiencing emotional distress and conduct problems during a prolonged disaster event. These findings are consistent with several studies suggesting that greater parental involvement and emphasis on family routines (but not positive parenting) predicted fewer child externalizing behaviors (Kelley, Palcic et al., 2010; Miki et al., 2019). In contrast, inconsistent discipline practices were predictive of more conduct difficulties for youths within this study, a finding supported previously by Miki and colleagues’ (2019) study of the Great East Japan Earthquake. Although we were unable to measure whether inconsistent discipline increased pre-to-post hurricane aftermath, it is conceivable that inconsistent discipline reported in this study fits within COR theory. Specifically, material, financial, and psychological resource loss spirals may impair parents’ abilities to enact discipline steadfastly and consistently, further distressing children (Hobfoll, 1989). These findings highlight the importance of reinforcing proactive parenting behaviors such as positive parenting and consistent responses to misbehavior in family-focused behavioral interventions following natural disaster (Kelley, Self-Brown et al., 2010). Consistent routines and structure provide children with feelings of safety and autonomy following a traumatic experience (Gershoff, 2002; Nordhal, Ingul, Nordvik, & Wells, 2006) and may be an important way for parents and children to cope together and improve overall family functioning (Piscitello, 2020; Prinstein et al., 1996).

In contrast to parental anxiety, which was significantly associated with children’s emotional symptoms, conduct problems, and hyperactivity/inattention, parenting practices were only related to conduct problems, as described above. It could be that within the short timeframe of data collection for this study, positive parenting practices (with regard to both positive parenting and inconsistent discipline) were secondary in influence to parents’ expressions and experiences of anxiety. However, before assuming this finding generalizes, there are some important contextual considerations. To start, unlike discipline inconsistency, positive parenting did not appear to be impacted by level of hurricane/flood exposure. As previously alluded, positive parenting behaviors may be relatively more stable over time and require less parental self-regulation to administer than consistent discipline under the regulatory strain of other post-disaster demands. This may in part explain why levels of self-reported positive parenting were quite high with relatively low variability. Even those who were in the lower range in this study still may have been within a generally effective range of praising their children for prosocial behavior. The high, restricted range might also be partly due to the single assessment timepoint, the sample’s higher-than-average levels of education, and the context of their participation. The fact that all participants completed a survey without any external incentive relatively soon after Hurricane Harvey might suggest that self-selection into the study sample biased toward those who model prosocial behavior and reward it in their children at higher-than-average levels. Future longitudinal studies with diverse samples should be conducted to generate greater understanding of how parental mental health reactions and parenting practices in the aftermath of disasters might influence their children’s psychological functioning over time, including those most *and* least affected by the disaster.

### Limitations and Strengths

While this study contributes to our understanding of parental impacts on children’s mental health following a weather-related crisis event, there are some limitations. This online survey was administered to a mixed sample of university students and employees as well as community members recruited through social media using snowball recruitment methods. These recruitment tactics may yield a sample that is more skewed in age, income, and educational levels; however, this methodology did allow for data to be collected quickly from a large sample immediately after Hurricane Harvey (within 1–3 months). Additionally, reports were retrospective from parents regarding their experiences during and after the hurricane. We were unable to assess the children directly, and thus, parent reports of children’s symptoms and functioning were purely indirect and subject to observer bias. Also, in cases where participants had multiple children, parents reporting on the child who was most affected potentially biased study results. It could be that collecting and analyzing data from children deemed least affected by parents might have shown protective effects of positive parenting practices. Further, information regarding marital status was not collected and may skew results due to protective factors that may be present in a two-parent household.

Additionally, our design and analysis precluded us from accounting for dynamic and bidirectional relations among parental characteristics, parenting behaviors, and child dispositional attributes and behaviors. For example, if a child acts out more under stress, then after a hurricane, might this increased frequency partly explain an increase in inconsistent discipline for a parent focused on reacquiring resources to ensure family survival and well-being? Relatedly, this study did not examine specific resilience factors, such as coping self-efficacy and parenting self-efficacy, or indicators of positive adjustment, such as well-being or post-traumatic growth, which can interact with and even transform post-disaster distress into valued meanings (Benight et al., 1999; Brickman et al. 2023; Long et al., 2020). Given that the study was cross sectional in nature, temporal dynamics among these variables and those we measured could not be examined, and causality cannot be inferred.

Despite these limitations, this is the first study to evaluate the various types of assistance offered to parents and the second to directly evaluate the potential influence of parents’ mental health on children’s mental health after Hurricane Harvey. Future studies are needed to assess the reliability and generalizability of these findings to parents and children who experience other disasters in various regions and cultures.

### Public Policy, Practice, and Future Research

These findings have considerable implications for informing public policy regarding government funding and disaster preparedness for families (Ronan et al., 2015). For example, in a time of collective crisis, individuals may lack friends or family who can help with transportation and evacuation, and this may cause greater vulnerability and distress. Notably, during disasters such as Hurricane Harvey, imminent need for social support often far exceeds its actual availability. Generally, after a crisis event, the primary task is to re-establish physical and psychological safety and the perceptions of physical and psychological safety, i.e., replacing or compensating for vital resource loss. In the current study, parents reported providing and receiving various types of assistance (e.g., evacuation, shelter, transportation, food, clothing, etc.). More research may be needed to examine the differential impact of instrumental types of assistance versus psychological assistance (Bistricky et al., 2023). Additionally, future studies should consider the impact of perceived effectiveness of assistance on child mental well-being. Given that parental depression and anxiety were both correlated with poorer mental health outcomes for youth (with anxiety being a significant predictor of child mental health outcomes), more focus may need to be given to ensuring parents are able to deal with acute, or potentially chronic, anxiety symptoms.

At broader systems levels, agencies are tasked with triaging assistance to those most in need of a variety of resources, and the historical effectiveness of agency relief to various communities may impact perceptions of threat to life following disasters (see Anderson 2008; Tynes et al., 2006 regarding inadequate relief response to African American communities in Hurricane Katrina). In the present study, minoritized racial-ethnic group status was not directly associated with parent or child mental health outcomes; however, minoritized status *was* modestly linked to greater perceived life threat in the midst of the disaster. Further, perceived life threat positively correlated with parent anxiety, which, in turn, correlated with several negative outcomes in children. Thus, minoritized status could possibly moderate these relations after disasters, possibly due to minority stress, both accumulated and disaster-history-specific (Meyer, 2003). Our findings are consistent with previous calls for agencies to better serve minoritized communities and combat systemic social inequities that maintain social vulnerability (Benevolenza & DeRigne, 2019; Bolin & Kurtz, 2018).

As noted, positive parenting practices were not predictive of child mental health in this study. Previous studies have found the importance of structured parenting practices for improving post-disaster coping and improved emotional regulation in children ((Lai et al., 2018a, b). Parents should be encouraged to create consistent routines with their children to facilitate adjustment and reduce escalating child conduct problems (Kelley, Self-Brown et al., 2010). However, in this study, a third of parents self-reported symptoms consistent with a likely depressive disorder or anxiety disorder, which may have impacted their parenting or their reporting of their parenting practices. More research and programs should be devoted to facilitating and supplementing effective parental practices in the aftermath of disaster, when typical parenting may be adversely affected, and children have increased needs.

Consistent with previous research on parent mental health and parenting practices following Hurricane Harvey (Elkins et al., 2021; Kelley, Self-Brown et al., 2010; Lai et al., 2018a, b), our findings suggest that parental anxiety might increase emotional dysregulation in children and worsen child conduct problems and hyperactivity-inattention symptoms. Although our findings are supported by a growing body of research, they must be interpreted with caution. In other words, pre-existing parental psychopathology and/or poor parenting practices might account for this relationship and exposure to Hurricane Harvey could serve as a moderating factor for this effect (Vigna et al., 2009). In addition, temporal research may inform our understanding of possible bidirectional relationships between parenting practices and youth behavior post-disaster (e.g., youth emotional symptoms and conduct problems may prompt subsequent inconsistent parenting practices), and how these variables may continue to shape each other over time. Future studies are needed as well that compare these results by age of children.

## Conclusion

This study examined the types and forms of assistance that parents and their children received during and after Hurricane Harvey. In addition, we examined parental mental health symptoms and parenting behaviors in relation to children’s mental health after Hurricane Harvey. Over half of parents received some form of assistance, which included food, water, shelter, and transportation. Further, parents reported use of some positive parenting strategies and minimal inconsistent discipline, yet higher inconsistent discipline was related to greater child conduct problems. A third of parents also reported symptoms indicative of clinically significant depression and anxiety. These and other parental factors accounted for a large amount of variance in child emotional symptoms, conduct problems, and hyperactivity-inattention symptoms. In conclusion, family and broader systems-level interventions supporting effective parent and child emotional responding might serve as a necessary component to post-disaster adjustment.

On behalf of all authors, the corresponding author states that there is no conflict of interest.
